# A Treatise on Diseases of the Rectum, Anus, and Sigmoid Flexure

**Published:** 1893-02

**Authors:** 


A TREATISE ON DISEASES OF THE RECTUM, ANUS, AND SIGMOID FLEXURE. By JOSEPH M. MATHEWS, M. D., Professor of the Principles and Practice of Surgery, and Clinical Lecturer on Diseases of the Rectum, Kentucky School of Medicine ; Visiting Surgeon, Sts. Mary and Elizabeth Hospital ; Consulting Surgeon, Louisville City Hospital ; Consulting Surgeon, Jennie Cassady Free Infirmary for Women ; late President Mississippi Valley Medical Association ; President Louisville Clinical Society ; Vice-President Louisville Surgical Society ; Member International Medical Congress, American Medical Association, Southern Surgical and Gynecological Society, Kentucky State Medical Society, State Board of Health of Kentucky ; Orator of the American Medical Association on Surgery, 1891, etc. With six chromo-lithographs and numerous illustrations. Octavo, pp. xvi.—537. New York : D. Appleton & Co. 1892.

In the preface to his book Dr. Mathews states that the work is the result of a desire to record his individual experience of fifteen years as a rectal specialist, and even a hasty perusal of the volume convinces one that his desire was a laudable one, for the work is an eminently practical and useful guide to the study and practice of rectal surgery. The text bristles with proofs that the author has had a large and varied practical experience upon which to draw for interesting material in the production of the volume before us. It contains, as he says, many things that are new to a work of the kind, among which may be mentioned the chapters on Disease in the Sigmoid Flexure, the Hysterical or Nervous Rectum, Anatomy of the Rectum in Relation to the Reflexes, Antiseptics in Rectal Surgery, and a New Operation for Fistula-in-ano. In the introductory chapter the author deals incidentally, but very intelligently, with the interesting subject of questions relating to examinations for life insurance of applicants who are or have been subject to rectal disease.

In Chapter III. the subject of constipation receives its full share of attention, and is exhaustively treated from the surgical standpoint. The author is certainly deserving of the thanks of the profession for the very proper and forcible manner in which he calls attention to the great importance of the external sphincter muscle as a factor in this common and distressing condition.

Antiseptics in Rectal Surgery is fully discussed in the fourth chapter, and the subject of Anesthesia, both local and general, is also alluded to. The use of cocaine is touched upon, and, from the author's remarks, we are forced to the conclusion that his experience with it in rectal disease, tallies well with that of most rectal surgeons respecting the usefulness of this drug as a local anesthetic, in that it does not seem to be of much value in rectal disease unless used in doses which might perhaps prove dangerous. In this chapter a few cases are cited where operations of considerable magnitude were performed under the anesthetic influence of whisky. In the chapter devoted to the treatment of Hemorrhoids several of the best methods of operation are described in detail, and the author rightly condemns that unscientific and unsurgical method of treatment largely resorted to by the charlatan, viz., "The Carbolic Acid Treatment" by injection. Perhaps, he is a little too severe in his condemnation of the "Whitehead Operation," although there is a vast amount of sound surgical argument in his seven objections to the method.

In Chapters VIII. and IX. the Diagnosis and Treatment of Abscess and Fistula of the Ano-rectal Region are fully discussed. The author is to be commended for the emphatic manner in which he condemns those surgeons (?) who refuse to operate on a fistula unless they always succeed in finding the internal opening of the sinus. It is certainly arrant nonsense to refuse to operate on these cases unless this opening can be found, for it cannot be found in one-half of the cases in which operative and radical procedures are an imperative necessity, and it is a matter of little consequence whether it is found or not, if the one who is to operate knows his business, as he ought. An operation is certainly indicated in the large majority of cases of fistula, internal opening or no internal opening, and if such opening cannot always be found, the skilful surgeon can quickly make one, which he must do, in order to cure his patient.

We are disappointed, however, at the brevity with which the author deals with the treatment of rectal abscess, and we certainly think he is in error when he says that the majority of abscesses in this region commence in the pelvi-rectal space of Richet.

The diagnosis and treatment of abscesses of the ano-rectal region are of great and lasting consequence, for upon their prompt recognition and proper management depends, to a great extent, the prevention of that disagreeable malady, fistula-in-ano. The author's treatment for these cases is commendable, so far as it goes, but it does not go far enough. We believe no surgeon is justified in treating an abscess of the ischio-rectal variety, or one originating in the pelvi-rectal space of Richet, who does not put his patient under an anesthetic and stretch the sphincter muscle, thereby putting the parts at rest, in order that the abscess cavity may have a proper chance to heal up without the formation of a fistula. This desirable result will not be obtained, in a large percentage of cases, unless this necessary step in the operation is performed ; otherwise the treatment outlined by the author is above criticism.

There is no doubt but that the author is correct in claiming priority in the method of treating fistula by the fistulotome, and it is gratifying to notice that he assigns the use of the ligature to its legitimate and proper sphere, viz., in sinuses running high up the bowel, where large vessels are liable to be involved, and where it would be difficult to control the hemorrhage if these vessels were incised. In the chapter on Strictures of the Rectum, the author differs widely from many authorities regarding the part played by syphilis and chancroidal disease in the production of strictures, but time and space prevent our going into detail regarding these differences.

In conclusion, it is a satisfaction to say that Prof. Mathews is to be congratulated on the production of his work, for, on the whole, it is a valuable, safe, and practical guide to follow, well worthy the careful perusal of any surgeon, young or old, who is at all interested in the particular branch of surgery to which it is devoted.

The author is to be congratulated also upon the efforts he has made and is making to wrest the treatment of rectal diseases from the domain of quackery, which has long tried to monopolize it, and place it in its legitimate sphere among the well-recognized specialties in surgery.

The work is a fine sample of the book-maker's art, and abounds with many handsome cuts and chromo-lithographs, which greatly add to its beauty and enhance its usefulness. E. C.

